# Training needs for staff providing remote services in general practice: a mixed-methods study

**DOI:** 10.3399/BJGP.2023.0251

**Published:** 2023-12-29

**Authors:** Trisha Greenhalgh, Rebecca Payne, Nina Hemmings, Helen Leach, Isabel Hanson, Anwar Khan, Lisa Miller, Emma Ladds, Aileen Clarke, Sara E Shaw, Francesca Dakin, Sietse Wieringa, Sarah Rybczynska-Bunt, Stuart D Faulkner, Richard Byng, Asli Kalin, Lucy Moore, Joseph Wherton, Laiba Husain, Rebecca Rosen

**Affiliations:** Nuffield Department of Primary Care Health Sciences, University of Oxford, Oxford, UK.; Nuffield Department of Primary Care Health Sciences, University of Oxford, Oxford, UK.; Nuffield Trust, London; Health Education England, London, UK.; Unit of Academic Primary Care, University of Warwick, Coventry, UK.; Nuffield Department of Primary Care Health Sciences, University of Oxford, Oxford, UK.; GP trainer, and MRCGP examiner, Ching Way Medical Centre, London, UK.; Health Education England, London, UK.; Nuffield Department of Primary Care Health Sciences, University of Oxford, Oxford, UK.; Nuffield Department of Primary Care Health Sciences, University of Oxford, Oxford, UK.; Nuffield Department of Primary Care Health Sciences, University of Oxford, Oxford, UK.; Nuffield Department of Primary Care Health Sciences, University of Oxford, Oxford, UK.; Centre for Sustainable Health Education, University of Oslo, Oslo, Norway.; Peninsula Schools of Medicine and Dentistry, University of Plymouth, Plymouth, UK.; Nuffield Department of Primary Care Health Sciences, University of Oxford, Oxford, UK.; Peninsula Schools of Medicine and Dentistry, University of Plymouth, Plymouth, UK.; Nuffield Department of Primary Care Health Sciences, University of Oxford, Oxford, UK.; Nuffield Department of Primary Care Health Sciences, University of Oxford, Oxford, UK.; Nuffield Department of Primary Care Health Sciences, University of Oxford, Oxford, UK.; Nuffield Department of Primary Care Health Sciences, University of Oxford, Oxford, UK.; Nuffield Trust, London, UK.

**Keywords:** e-consultations, general practice, knowledge, remote consultation, training needs, video consultations

## Abstract

**Background:**

Contemporary general practice includes many kinds of remote encounter. The rise in telephone, video and online modalities for triage and clinical care requires clinicians and support staff to be trained, both individually and as teams, but evidence-based competencies have not previously been produced for general practice.

**Aim:**

To identify training needs, core competencies, and learning methods for staff providing remote encounters.

**Design and setting:**

Mixed-methods study in UK general practice.

**Method:**

Data were collated from longitudinal ethnographic case studies of 12 general practices; a multi-stakeholder workshop; interviews with policymakers, training providers, and trainees; published research; and grey literature (such as training materials and surveys). Data were coded thematically and analysed using theories of individual and team learning.

**Results:**

Learning to provide remote services occurred in the context of high workload, understaffing, and complex workflows. Low confidence and perceived unmet training needs were common. Training priorities for novice clinicians included basic technological skills, triage, ethics (for privacy and consent), and communication and clinical skills. Established clinicians’ training priorities include advanced communication skills (for example, maintaining rapport and attentiveness), working within the limits of technologies, making complex judgements, coordinating multi-professional care in a distributed environment, and training others. Much existing training is didactic and technology focused. While basic knowledge was often gained using such methods, the ability and confidence to make complex judgements were usually acquired through experience, informal discussions, and on-the-job methods such as shadowing. Whole-team training was valued but rarely available. A draft set of competencies is offered based on the findings.

**Conclusion:**

The knowledge needed to deliver high-quality remote encounters to diverse patient groups is complex, collective, and organisationally embedded. The vital role of non-didactic training, for example, joint clinical sessions, case-based discussions, and in-person, whole-team, on-the-job training, needs to be recognised.

## Introduction

*The NHS Long Term Plan*, published in 2019, commits to digital-first primary care (in which general practices must offer and promote online and telephone consultations) by 2023–2024.^1^ There is also growing emphasis on supporting patients with care navigation (including navigating digital access and consultations).^2^

In 2019, the NHS established a Digital Academy with a view to training a national cohort of clinical informatics leads.^3^ The ‘Topol Review’, published in 2019, emphasised that healthcare organisations would need a strong workplace learning infrastructure; a reputation for training and support; proactive rather than reactive learning activities; and dedicated staff time for development and reflection on their learning outside clinical duties, though it made no comment on how these changes might be achieved or resourced in primary care.^4^ It recommended training in (among other things) the skills to undertake remote triage, assessment of a patient’s digital literacy and suitability for particular digital modalities, remote and online consultations, and teaching and motivating others to use novel technologies in their work.

There are numerous stakeholders in the digital training space, including national professional, statutory, and regulatory bodies; educational providers within and outside universities; producers of digital competency frameworks and assessment tools; an annual cohort of ‘Topol Digital Fellows’; and provider organisations with significant experience using remote modalities, including out-of-hours services such as NHS24, the COVID-19 Clinical Assessment Service, charities, and private providers. GP trainees’ remote clinical skills are summatively assessed through (among other things) a portfolio of workplace-based assessments and (from autumn 2023) simulated video consultations, which count towards Membership of the Royal College of General Practitioners (MRCGP) and Certificate of Completion of Training (CCT). Regional and locality-based training are supported through a complex infrastructure, including: GP Vocational Training Scheme regional events; regional training hubs; primary care networks (PCNs); NHS England integrated care boards; NHS Scotland regional health boards; NHS Wales local health boards; local out-of-hours telephone training providers; practices; and peer educators, including fellow clinicians and ‘super-users’ of technologies.

**Table:** How this fits in

The introduction of remote triage and remote consultations has outpaced the training of staff to deliver these modalities. Qualitative findings on training needs from a large study in UK general practice and a review of peer-reviewed and grey literature are synthesised in this paper. We found many staff feel underprepared, not just for using new digital technologies, but also for conducting effective and safe encounters by telephone. A draft set of competencies for clinical and support staff at different levels is proposed.

There is much policy enthusiasm and activity around training for remote and digital NHS services. But visits to front-line general practice revealed that there was a substantial mismatch between the worthy aims of the Topol Review and the reality of current provision. In this study, we sought to explore this gap empirically and identify training needs, core competencies, and learning methods for different staff groups, which would help reduce this mismatch.

## Method

This was a mixed-methods study in UK general practice.

### Origin of this substudy

The substudy reported here emerged from the ongoing Remote by Default 2 (RBD2) study, whose protocol and baseline findings have been published elsewhere.^5^^,^^6^ RBD2 has three main work packages. The first is a longitudinal multi-site case study from 2021 to 2023 of how remote services are developing (or not) in 12 UK general practices across England, Scotland, and Wales (nine of which are either teaching or training practices), selected to represent a wide range of digital maturity from traditional (few digital services) to digital leaders (providing state-of-the-art digital services and supporting other practices), using interviews, ethnography, and some quantitative data. The second is co-design work with patients and staff to develop more patient-centred pathways for digital access. The third is national stakeholder engagement through elite interviews and multi-sector workshops.

Early interviews, ethnography, and workshops identified unmet training needs among both clinical and support staff.^6^ Notwithstanding some pockets of good practice, there appeared to be a mismatch between training needs and training provision. Accordingly, we created a subset of data for targeted analysis, comprising people’s descriptions of training they had received (or would have liked to have received) alongside reflections from trainees and trainers (clinical and non-clinical) on training needs and preferred learning methods. These data were supplemented with additional material. See Box 1 for a summary of data sources and responder numbers.

**Box 1.** Summary of data sources

**Source/type of data**	**Nature of full dataset**	**Subset of data analysed for this article**
Longitudinal case studies of general practices (RBD2 study^5^^,^^6^)	Field notes from ethnographic visits, and staff and patient interviews in 12 UK general practices followed for 30 months. Documents such as websites and leaflets. Text data transcribed and coded on NVivo.	Interview data where staff indicated their learning or training needs. Field notes pertaining to staff members’ ability to arrange or deliver remote services. Total 44 pages of excerpts (representing 30 staff).
National stakeholder interviews (RBD2 study)	Semi-structured interviews with stakeholders from policy, industry, primary care strategy, and patient advocacy groups. Audio-recorded and selected sections transcribed.	Extracts of data from six interviews including two out-of-hours clinical leads, one GP trainer, two GP out-of-hours training programme directors, and one software provider. Total 15 pages.
Multi-stakeholder workshop (RBD2 study)	Online workshop (January 2023) with 51 participants including clinicians, support staff, local and national policymakers, and trainers. Video- and audio-recorded plenary sessions, and virtual breakout groups (relevant sections transcribed).	Interdisciplinary group discussions about perceived training needs and training experiences. Presentations by training providers (for example, out-of-hours general practice). Policymakers’ perspectives and expectations. Total 3 hours of discussion; eight pages of extracts.
Additional trainer and trainee interviews (service evaluation)	Interviews and participant observation by University of Oxford MSc student in collaboration with Health Education England to evaluate current training provision for GP trainees. Transcribed and coded on NVivo.	Interviews with 10 trainees and 10 trainers; field notes from five trainers’ meetings. Total >200 pages.
Training materials	Formal training programmes for students, clinicians, or support staff (curricula, handouts, and tutor and student workbooks). Tips and guidance from national and local peer educators, for example, infographics, slide decks, videos, and checklists.	Content and rationale of formal training, including target learners, learning outcomes, standards, and criteria for assessment. Scope and content of informal tips and advice. Total four undergraduate and 12 postgraduate courses, and two courses for support staff; 20 pages of informal materials.
Training provider comments	Brief online interviews or email exchanges with people supplying training materials. Not audio-recorded; brief contemporaneous field notes taken.	Notes and emails from 25 individuals (four national clinical leads for out-of-hours care, three postgraduate/assistant deans, three GP training programme directors, six medical school communications leads, two academics doing PhDs in remote consultations, one out-of-hours clinical lead, three additional GPs, and three representatives of professional bodies). Total 15 pages.
Research literature	One published systematic review^7^ plus one additional primary study (survey of allied professionals).^8^	Nature of training interventions that have been evaluated in published studies. ‘Before’ data on baseline competencies; ‘after’ data on satisfaction and learning outcomes. Discussion sections (for theoretical models of learning).
Grey literature	Recommendations of professional bodies^9^^–^^18^ and policy groups;^2^^,^^4^^,^^19^^–^^24^ Health Education England survey of training needs for remote services in 791 NHS primary care staff;^25^ and the General Medical Council survey of 18 434 trainer and 48 785 trainee doctors.^26^	Relevant sections of professional codes of practice, standards of excellence, and suggested curricula. Survey responses to closed questions about time available for training, priority topics for training, preferred modalities for training, experiences of wellbeing and burnout, plus free-text comments. Total 40 pages of excerpts.

### Extending the dataset

We extended the RBD2 dataset with a targeted literature search on training materials for clinicians and support staff, and a service evaluation of clinical training. We also searched for articles on training for allied professionals and support staff.

To obtain training materials for clinicians (and comments from those who developed or used these), we emailed a purposive sample of content experts, including: national clinical leads for video-consulting programmes in England (identified through Health Education England), Wales, and Scotland (identified through departments of health); assistant postgraduate deans for general practice in these jurisdictions; and leads for communication skills teaching in four UK medical schools (Cardiff, Leicester, Aberdeen, and Oxford). Responders were asked to suggest others to contact. We also invited contributions via social media (Twitter, and the Facebook groups: GP Survival; GP Survival Wales; GP Survival Scotland; and Resilient GP) and email networks (covering urgent care in UK and European out-of-hours GPs). We used a Google search to identify materials offered by professional bodies. Training materials for induction of support staff were obtained from practices in the RBD2 study.

Health Education England provided headline findings from a national survey of 791 primary care staff (*n* = 491 clinical);^25^ we also conducted the 2022 General Medical Council survey of trainer and trainee doctors.^26^ Health Education England invited the authors to undertake a rapid evaluation of training provision for GP trainees. To that end, one author interviewed 10 trainers and 10 trainees, and attended five meetings of a policy group at Health Education England between November 2022 and May 2023. Interviews were audio-recorded and contemporaneous notes made in meetings.

### Theoretical framework

Our theoretical starting point was that learning to provide and support remote care requires people to develop complex forms of knowledge and apply that knowledge, both individually and as part of multi-professional teams, in unique, potentially stressful, and unpredictable real-world situations. We drew on several theoretical perspectives: experiential learning;^27^ competence and capability;^28^^,^^29^ social learning theory;^30^ how leader–follower relationships shape learning;^31^ sociomateriality;^32^^,^^33^ and organisational-level theories of complex knowledge^34^ and routines^35^^,^^36^ (Box 2).

**Box 2.** Theoretical frameworks informing this study

**Experiential learning**
Adults (like children) are generally highly motivated to learn; they learn things that are immediately useful to them; and they learn best in a self-directed, task-oriented, and experience-based manner.^27^ The adult learner integrates new experiences into existing conceptual models, modifies these models in the light of experience, and tests the new models against external reality.^27^ This theory is helpful but not sufficient in guiding training, since (for example) feedback from others is an important component of the learning process^37^ and novice learners in particular may overestimate their competence and fail to recognise key learning needs.^38^
**Competence and capability**
*Competence* is defined as *‘what individuals know or are able to do’*; *capability* as the *‘extent to which* *individuals can adapt to change, generate new knowledge, and continue to improve their performance’*.^28^ While competences are inherently conservative (for example, knowledge of an existing evidence-based guideline), general (apply to multiple situations), and assessed retrospectively (for example, by a trainee presenting a portfolio of evidence), capabilities relate to the creative production of *new* knowledge in *particular* situations — for example, generating multiple possible diagnoses and working through different management scenarios when assessing a complex case.^29^ Related to capability is *entrustability*, defined as occurring when clinicians can trust a trainee or colleague to perform a particular role unsupervised. Examples of entrustable professional activities include seeing undifferentiated patients in person, doing telephone consultations, and being duty doctor.
**Social learning theory and relational aspects of learning**
Learning to use technology is a highly *social* activity, as people learn by observation (from watching others), collectively problem solve, and share stories about troubleshooting.^30^ Indeed, multimedia storytelling in groups can be a powerful learning tool.^39^ In work-based learning, a positive relationship between the leader (trainer) and the follower (trainee) leads to higher expectations and better performance — the so-called Pygmalion effect.^31^
**Sociomateriality**
The material properties and affordances of technologies shape and constrain what humans are able to do in a given context. Technology is designed for an *idealised* system (‘work as imagined’), but staff in any organisation must learn or develop workarounds (articulation practices) to deliver ‘work as done’.^33^ Contemporary learning is often more about making wise decisions in particular contexts, constrained by materiality, than about acquiring abstract knowledge.^32^
**Organisation-level theories of complex knowledge and routines**
Tsoukas’ notion of *‘complex* [organisational] *knowledge’*^34^ emphasises that knowledge in organisations is collective as well as individual, and embodied in business-as-usual patterns of acting and interacting. An organisational routine is defined as a repetitive pattern of interdependent action involving multiple actors.^35^^,^^36^ As experienced organisational actors, people ‘know the ropes’ — and usually know more than they can tell. People new to an organisation need to learn those ropes (for example, even if they know how to use a technology *in general*, they need to learn how it is used in the organisation). These distributed views of knowledge align with Gabbay and le May’s notion of mindlines: collectively generated and shared understandings that evolve through discussion and shared practices.^40^

### Data management and analysis

The wider RBD2 dataset, at the time of writing, was being collected over 27 months and entered and broadly coded on NVivo software. Extracts relevant to training were compiled into a Microsoft Word document. These were read and re-read to gain familiarity, before constructing and iteratively refining a more specific coding framework covering aspects of teaching, learning, and related concepts (such as ‘context for learning’ and ‘organisational and system knowledge’). We developed an analytic framework in Microsoft Excel (preferred by the lead author because of its familiarity and manipulability) and extracts were charted from our various data sources, further refining the framework as we added each successive source. The framework was reviewed and improved by the other authors. These descriptive data were then theorised by applying the theoretical lenses described previously.

A set of outline competencies for providing remote primary care services covering different student and staff groups was developed through repeated iteration and discussion among the RBD2 research team (which included two GP trainees who had recently completed their CCT, two experienced GP trainers, and one policy lead). A draft set of findings, including these outline competencies, was written up in an interim document. This was shared with researchers in the wider RBD2 research team (who had close familiarity with one or more participating practices) and with selected key stakeholders (national teaching or training leads, clinical trainers, clinical trainees, and trainers of reception and support staff), as well as with three lay members of the RBD2 advisory group. The synthesis was refined in light of their feedback.

## Results

### Overview of dataset

The subset of data analysed for this article is shown in the last column in Box 1; the middle column shows the wider datasets from which these data were selected. The search for training materials combined with relevant stakeholder interviews for RBD2 identified a total of 25 individuals involved in training. These individuals supplied a wide range of training materials targeting all career stages from undergraduate level to established GPs and practice teams across England, Scotland, and Wales. In the sections below, we illustrate our findings with representative quotes, using pseudonyms to protect responder identities. Additional quotes can be found in Supplementary Box S1.

### The current context for training

Our ongoing ethnographic research, which started in September 2021, revealed severe — and in many cases, unprecedented — pressures from high workload and staff shortages:

*‘We* [reception] *are a bit short staffed and we do get a lot of temps. What we try to do is look for long-term temps who are going to stay for three to six months. It takes two weeks to train a receptionist up and then she leaves, and when I’m doing that training I put my other work to one side. It’s very deflating when you’ve put a lot of time and effort into training a receptionist and then she leaves. Sometimes they leave if they live far, because the travel, it’s just too much.’*

(Lucy, lead practice receptionist, England)

While interviewees acknowledged a need for training in remote service provision, in reality, the time, headspace, and resources for such training were very limited:

*‘I was hired specially for chronic disease monitoring. But secondary care are so overstretched, the workload coming in from them means I’m always fully booked. I don’t have time to do the training — to learn the templates or to use remote consults — let alone actually do the LTC* [long term condition] *reviews.’*

(Oliver, GP trainer, Scotland)

Abuse from a small minority of patients was identified as a significant contributor to stress, resulting in high support staff turnover and thus a heavy workload of inductions for new staff. Many training opportunities had been lost in the pandemic:

*‘It’s been terrible for their training. Really bad. I don’t think they* [trainees] *would say it’s as bad as I think but they have lost a lot of experience* […] *They haven’t had as much clinical exposure to physical signs, to clinical examination. Telephone triage, we used to not let them do it until they were really experienced. And they were day one, ST1, the first day in general practice doing telephone triage.’*

(Sheena, GP trainer and associate dean, England)

Stressed staff often lost both motivation for, and receptivity to, training:

*‘I tell trainees you’ve got to be curious, but they’re under pressure so they lose their curiosity.’*

(Ravi, GP trainer and course organiser, England)

These findings are supported by the Health Education England survey, which found that many administrators, practice managers, GPs, and nurses had little or no protected time for training;^25^ the General Medical Council survey of trainee and trainer doctors, which identified rising levels of stress and burnout, and a proportion of trainees unable to use all their allocated training time;^26^ and a survey of allied professionals, which identified numerous unmet training needs.^8^

Every individual remote encounter can be viewed as part of a complex process of ongoing care (for example, a phone call must be booked, documented, and followed up). The vast majority of remote encounters in this study occurred over the telephone. Staff needed to adjust their communication styles, rapport-building, information-giving, and safety-netting practices to accommodate the remote modality. They also needed to work quickly, using a variety of technologies (both old and new) to document findings and decisions, book (further) appointments, and cross-refer to other members of the practice team. These workflows and interactions were often complex, and were shaped and constrained by historical divisions of labour within the practice and by the material properties and limitations of available technologies, which varied from practice to practice:

*‘I know AccuRx have good videos and they do training. But there’s two things you need to know. One is how it works and one is how it works in OUR workflow. So we can’t just say to AccuRx, can you come and train us in how to use it? Because they don’t know our workflows.’*

(Jane, practice manager [workshop participant], England)

A decision to offer a particular modality of appointment was often clinically, logistically, and ethically complex: the person (or people) making the decision had to take into account capacity and staffing constraints (for example, only five available face-to-face appointments on a day), the clinical need (and associated uncertainties) of the patient on the call, the wider needs of other patients who were seeking (or who may require) care, practice policies for supporting patient choice, and the consulting preferences of the individual practitioner. The contextual and organisationally situated nature of remote triage in particular means that generic, ‘standardised’ training may be useful for gaining *competence* in using new technologies (for example, understanding the functionality) but of limited use in developing the *capability* to use these technologies adaptively, collaboratively, and under pressure in the real world of general practice.

### Perceived training needs

Desire for further training was almost universal. Clinicians identified four broad training needs:

- technical skills (how to use a remote technology);
- communication and clinical skills (how to communicate effectively using a remote technology);
- implementation skills (for example, how to embed remote encounters into practice work and mitigate digital inequalities); and
- pedagogical skills (how to train other staff or patients).

Where a new digital technology was involved, training was perceived to be essential and greatly valued. Staff training was often part of the package when purchasing digital technologies.

Many novices and some more experienced staff disclosed that they knew how to use digital technologies but not confidently or at the required pace. New trainees who had not yet undertaken remote consultations prioritised the need to learn technical skills (for example, using video technology), relevant regulations (for example, information governance standards), and the basics of safe practice (for example, avoiding never events):

*‘I’d maybe feel like I needed some training* […] *about the logistics and the sort of consent and confidentiality aspects of video recording because obviously our telephone calls in the practice are all recorded* […] *so from a sort of storage point of view, in terms, I know it’s all stored and the confidentiality aspect … ’*

(Bob, novice GP trainee, Wales)

While some were very aware of their own limitations, others felt they had quickly acquired skills and the confidence to take on greater risk and responsibility after being *‘thrown in the deep end’* (Jeremy, newly qualified GP [workshop participant], England) in the early months of the pandemic — perhaps reflecting the Dunning–Kruger effect where naïve learners overestimate their competence.^38^ Trainers were more circumspect and described the current cohort of trainees as having missed out on closely supervised clinical experiences (both traditional and remote) during this period, sometimes with safety critical consequences, particularly in relation to telephone consultations:

*‘I’m seeing a lot of young doctors skating on thin ice.’*

(Paul, GP trainer and examiner for Royal College of General Practitioners’ Remote Consultation Assessment [RCA] examination, England)

*‘I had four* [complaints] *… People not feeling heard. People feeling fobbed …* [one trainee] *missed somebody with metastatic cancer … And so, I’m quite risk-averse with telephone.’*

(Dorothy, GP trainer, England)

They described slow pace and poor judgements by trainees sifting triage calls as contributing to inefficiency through double-handling and loss of continuity:

*‘A triage session is fast paced and intense. Decision-making speed is linked to clinical confidence and experience (including familiarity with local pathways). It means the more junior* [staff members] *can’t handle peak demand times* […] *Remote triage done badly means unnecessary telephone appointment or duplication of telephone appointment plus subsequent face-to-face appointment. Remote triage done well has resulted in faster responsiveness, greater ability to stream to right clinician for task, and preservation of continuity.’*

(Ramesh, GP and GP Federation Chair, England)

Experienced trainees and established clinicians talked about their desire to achieve excellence (both communicatively and clinically) in the remote encounter, and transfer the clinical skills and experience acquired in traditional consultations to the remote setting:

*‘We need to distinguish between the* minimum *threshold of “adequacy and safety” and the* desired *threshold of “attunement, attentiveness, and holistic care”. How do we make training pathways … I think it’s impossible to quantify the skill of attentiveness but at least we could describe it and think about how we could build that into training and – potentially – accreditation for GP registrars?’*

(Fiona, newly qualified GP [workshop participant], England)

*‘It took me while to get used to consulting over phone. When used to seeing people* [face-to-face]*,* [and how we were] *taught at medical school, so much of your consultation is visual. I can tell a sick child within thirty seconds of walking into the room. Also my treatment for a child with viral infection is me examining that child and reassuring mum nothing needs to be done. So that is really tricky over the phone, having to ask so many questions, and reassurance for mum is almost impossible* […] *when you haven’t examined them.’*

(Suzanne, GP trainer, Scotland)

Interviewees with lengthy experience of training clinicians in telephone consulting emphasised that basic listening and core history-taking skills were crucial for safety and quality:

*‘*[Clinicians need to] *think about the words they use when they’re speaking to you. The patient’s level of concern. The time of day. These basic things. In the overwhelming majority of “learning events”* [for example, near-miss or critical events] *people got the basics wrong. If you get the basics right then it’s solid.’*

(Farida, senior GP working in out-of-hours service, England)

Support staff also wished to gain knowledge and skills in the technologies essential for their roles, efficient and safe assessment of patient suitability for remote modalities, and safeguarding. They recognised that triage sometimes involves making urgent and far-reaching clinical decisions for which they felt under-trained:

*’I think as reception staff, it is a little bit tough to triage. I think that it gives the doctors a bit of a break, but at the same time, I feel it’s a little bit pressure. Say I get it wrong? That’s all I worry about … when I first started here, a lady called and said that her baby had a blocked nose, so I was like, “Oh, he’s got a blocked nose.” Now, I didn’t think anything about it, because it’s just a blocked nose. But then two hours later, the baby was in a really bad way and then … so that plays on my mind because to me, “He’s got a blocked nose.” But I don’t know that a blocked nose is a serious problem in a baby, you know? It’s stuff like that.’*

(Priya, receptionist, England)

They considered communication skills (which were widely described by support staff as ‘people skills’ or ‘customer care’) essential for all patient-facing staff. Many support staff also identified the need to train in how to use technology in the context of (often complex) practice workflows and interactions between different staff groups (a domain that few clinicians brought up), and how to negotiate with patients when their preferred modality was unavailable.

Published research^41^ and the Health Education England survey^25^ confirm that the confidence and knowledge of students, trainees, and established clinicians prior to training in remote and digital encounters is often low. Training budgets were tight and not always awarded even when in people’s contracts; they varied by locality among salaried GPs, allied professionals, and support staff.

### Participants’ experiences of training

Training provision varied considerably across our sample of 12 general practices. Some offered formal training linked to locality-wide digital capacity-building initiatives:

*‘I feel we had a good level of support. Obviously, AccuRx was provided by the CCG* [clinical commissioning group] *and I’m actually just remembering another digital suite that we recently had access to which is Ardens* […] *And they’ve, even more recently, given us access to a dashboard where we’re able to see all the QOF* [Quality and Outcomes Framework] *targets and achievements. So, all of this has been provided by the CCG. If there are any issues with the programs, with the software, obviously, we can contact their IT team. We were provided with all brand-new monitors that actually have in-built cameras which will then allow us to conduct video consultations and remote meetings.’*

(Isaac, GP trainee, England)

Others had no dedicated time for training or offered limited training to some but not all staff:

*‘There’s nothing formal. It’s all on the job.’*

(Mel, assistant practice manager, Scotland)

Senior and digitally confident staff tended to take up this training and then train others, with some training providers developing this into a ‘digital champion’ and ‘super-user role’:

*‘Before we had this AnyConnect, or I can’t even remember what it’s called now* […] *there was kind of an online training thing that you could do and I think perhaps three or four of the staff might have done it* [laughs] *but yeah the doctors … I mean we said to the doctors you do it first and then if you think it’s any good then we’ll roll it out to everybody else. But they weren’t that keen and like I say* [GP name] *was the only keen one.’*

(Lesley, practice manager, Wales)

*‘“Digital champions” are the network of “super” digital users that we upskill – either in practice or across PCNs and we say that’s the legacy that we leave behind. We have this experience from* [names two PCNs]*. I personally feel this is a better use of resource than employing “change managers” who are often not embedded in practice but are given “commissioning priorities” to implement such as online consultations.’*

(Bernadette, ex-NHS manager, now head of private-sector company offering training and support for digital primary care, England and Wales)

Some clinicians had never been offered training in using the telephone for clinical assessment — a finding that was affirmed by the results of the Health Education England survey^25^ and by an earlier UK-wide survey of GP trainees.^9^

Staff described various types of learning and training, most commonly mandatory inductions for new staff and self-study e-learning resources (often technology focused and provided by the technology’s supplier). Certified e-learning courses were popular with national policymakers and regulators across the three nations but viewed as (at best) a superficial and partial solution to the training needs challenge:

*‘CQC* [Care Quality Commission] *inspections and the proliferation of commercial providers combines to drive this focus on certifiable e-learning, which keeps mushrooming.’*

(Eleanor, GP tutor [workshop participant], England)

Trainees felt they learnt a lot from shadowing experienced clinicians undertaking telephone consultations, and by having one-to-one follow-up sessions after e-learning focused on a digital technology to explain how to use the technology within practice workflows:

*‘I learnt a lot from shadowing my trainer and other GPs, learned about how to ask certain questions, and about the importance of finding out the “back story” to a phone consult. I think this made me less “transactional”.’*

(Roger, GP trainee, England)

Indeed, staff undertaking new technology-mediated roles often perceived that training in using the technology *to do the job* had to happen on the job:

*‘I just said, ”No, no, no I’m not doing anything like that* [learning difficulty reviews] *until somebody can sit and do things in front of me, show me what things I have to click, what I have to ask”, or I’d like to actually observe them being done. Some people learn better by observing something being done, taking notes. “Okay so she’s asked him about that and that made that difference” or something that I wouldn’t think to do.’*

(Bruce, paramedic background, now working on long-term condition reviews, England)

When a new technology was introduced, staff who knew how to use it informally trained and supported others, and such assistance transcended traditional hierarchies (for example, we observed doctors asking receptionists how to use a new telephony system, and a clinical assistant training GP partners in a new online system).

These findings contrast with Health Education England’s survey, which found that *‘e-learning’*, *‘face-to-face training’*, and *‘live webinars’* were the most popular options selected from a tick list, but options such as shadowing or on-the-job training were not available to choose.^25^ Equivalent data for the other national health bodies were unavailable.

### Training providers’ views on effective training methods

None of the clinical trainers interviewed mentioned classroom teaching or self-directed e-learning except to raise concerns about their limitations. In common with trainees and other clinical staff, trainers had positive views on the value of shadowing, debriefs, case-based tutorials, on-the-job training through real-time ‘just ask’ opportunities, and facilitated case discussions in groups:

*‘I have my trainees listen in on me as an experienced GP, and they’re like, “Well, you know, how do you do that?” It is something that just comes with lots of practice. Yeah. And, and yeah, you know, to be able to have a phone call* [with] *several mental health patients that you’re not seeing face-to-face, but a totally spilling out all their deepest, darkest secrets to you. It’s quite the skill to get them to feel comfortable to do that. Over the phone.’*

(Jennifer, GP trainer, England)

*‘We’re all fallible. We* [trainers] *talk them* [trainees] *through cases* [of telephone consultations]*, surface where the trip wires are* […] *It’s not just GPs, it’s social workers and other professionals too. Everyone bring a case, talk through them, name your fears. We need to shift people from “fear-based medicine” to “courage-based medicine”’ where you’re doing what’s right for the patient. We discuss those cases both experientially and academically, though there’s not much on it in the academic literature. We also have a video-based training platform where we teach people how to build rapport over the phone, how to recognise when you’re losing rapport, how to build it up again. This all happens by sharing patient stories. And listening in to calls.’*

(Farida, senior GP and experienced trainer working in highly regarded out-of-hours provider, England)

Undergraduate teachers commented that shadowing in the remote environment was logistically more difficult than in-person shadowing, and sometimes met technical barriers (for example, the speaker phone not working).

Providers of training for remote triage and consultations emphasised that specific, detailed, and timely feedback on actual encounters and decisions was a particularly effective way of achieving learning and improving performance, affirming Dehaene’s work on the role of feedback in learning.^37^ The following quote is from a leading provider of out-of-hours services delivered mostly by telephone:

*‘When patients have a phone consultation they usually get told “this call may be monitored for quality and training purposes” but in reality it’s rarely used for either of those things, it’s only even listened to for defensive reasons after someone complains! If we can pick up doctors’ learning needs early, we can help them understand what their peers are doing and why and how they’re an outlier.’*

(Sandra, clinical director of out-of-hours service where experienced doctors routinely audit telephone consultations, England)

This provider had introduced a clinical coordinator role — an experienced GP who was available in real time to help clinicians with difficult cases and who also scanned the records of completed calls to identify examples of practice that could be improved. All clinicians received feedback on their calls, with the vast majority being told that their decisions were appropriate. For the remainder, a non-judgemental, supportive approach was taken, with the senior colleague shadowing their calls and providing case-based, one-to-one training.

Support staff training was described by several interviewees as somewhat didactic, focused on specific digital technologies, and occurring mainly at induction of new staff. The following quote illustrates the important additional value that was achieved (in many but not all practices) from on-the-job training:

*‘Senior receptionists get trained and pass that on to juniors. Every new receptionist gets an induction, and then they’ll shadow another receptionist at the beginning so they get up to speed. Not being big headed but I feel I’m really good at training up other members of the team. If something new comes in, a new system or a new process, I’m forever sending out emails and WhatsApps with the step-by-steps. And they’re not afraid to come back and ask if they feel they need help, and that’s a good thing. I tell them nothing’s too small, just ask. In my eyes, that’s what I’m paid for, I’m paid to support my team. I tell them, even if you ask the same question 100 times, it doesn’t matter that’s what I’m here for. We’ve got templates for everything on there, and sometimes it’s just they need to find the wording to find the template for what they want to do.’*

(Caroline, lead practice receptionist, England)

On-the-job training occurred both through formalised periods of shadowing and via informal methods such as internal emails with tips, resources, and invitations to *‘always ask’* (Caroline, lead practice receptionist, England) a senior colleague who made herself accessible and created a non-judgemental culture.

Our ethnographic observations affirmed the important but often hidden role of informal interactions in the workplace in helping staff acquire confidence to use (and, especially, troubleshoot) technology.^30^ One GP from a digitally advanced practice commented that, even though working from home was feasible technologically, this option was only used occasionally and *ad hoc* because the benefits of peer support and supervision when on-site outweighed any convenience advantage of homeworking.

### Towards a set of competencies and capabilities for remote encounters in general practice

Our analysis of undergraduate training materials revealed that the curriculum now includes basic principles of remote consulting under ‘communication skills’. Postgraduate training schemes (as well as more informal ‘tips and guidance’ prepared by peer educators and professional bodies) sought to prepare trainees for the RCA examination and CCT milestone. Formal training courses for support staff emphasised technical competence, efficiency, and safety of triage decisions.

Almost all the formal training resources and programmes we identified were aimed at individuals and focused on isolated individual encounters (the one-off triage decision or remote consultation). Most did not fully address — or even acknowledge — the challenges of achieving coordination of a care journey that would likely unfold over time and be distributed across a multi-professional team:

*‘There is no point doing training on products without looking at the practice processes that need to sit behind a remote methodology. For example, let’s say a man wants a prostate check, before the patient would have come in for an appointment, now they* [the practice] *text him a link to a patient information leaflet, fill in the blood request, and tell them to make a blood appointment if they want to proceed, only seeing them once the result is back. But this process needs to be formalised to be effective* […] *and you need to build in risk management and performance management.’*

(Graham, GP trainer and out-of-hours doctor, England)

*‘I was speaking* [at a conference] *about continuity of care – something I call “micro-continuity”, by which I mean* through *the acute episode. I described an example of calling back an anxious old lady with palpitations and slightly high BP* [blood pressure]*. That call-back probably prevented a trip to A&E* [accident and emergency]*. You’ve got to give the patient a sense of “being held”.’*

(Sarah, director and trainer in an out-of-hours service, England)

Several interviewees emphasised the importance of a collective and shared understanding of these complex processes, and staff who had been involved in whole-team training (often pre-COVID-19) greatly valued it:

*‘A whole team understanding of how to do something is important without a demarcation of “clinician” versus “non-clinician”.’*

(Mary, training provider [workshop participant], England and Wales)

*‘What we used to do before COVID we would have what we called protected education time which was funded by the health board and we could get all of the practice together for an afternoon and then the health board would take the telephone calls and we used to split it. Sometimes we’d do* [um] *training for everybody, we’d all be together in the same room and other times we might have clinicians doing something and then the admin staff doing something and that was really good, and it was really valuable, and we really enjoyed it. We used to get a nice lunch and we’d all share lunch and it would be nice but we’ve not been able to do that since COVID and I think, I think that has impacted … there’s the staff all say, “When are we gonna have our protected educations back again?” you know, because I think it helped, it might not even be the subject of what you were talking about but somebody might say “Ah, you know, I couldn’t do this the other day”, and then somebody else will say, “Oh, well I had that problem the other day and, you know, I was able to do this* [to solve it]*”, and it was sharing then, you know?’*

(Tegan, practice manager, Wales)

One contributor commented that whole-practice team training was not merely about learning complex work routines but also about developing and negotiating the shared values and ethical principles that underpinned decisions about how complex patients were managed and how technologies were deployed in practice:

*‘Continuity of care, I’m thinking of vulnerable families, people with MH* [mental health] *conditions and their families. A moral compass needs to be injected into training to encompass these issues. Conversations need to be held about local values with staff members to inform training. What is our purpose, what do we care about here?’*

(Rosemary, education lead at arms-length body [workshop participant], England)

Supplementary Table S1 and Supplementary Table S2 (downloadable format) list some outline competencies and capabilities for delivering remote services by different staff groups and different levels of seniority, based on our synthesis of the published literature, empirical findings, and feedback from an online workshop of trainees and trainers. Training for support staff is briefly covered for completeness but will be discussed in more detail in a separate article.

## Discussion

### Summary

This mixed-methods study in UK general practice has shown that learning to provide remote encounters occurs in a context of high workload, understaffing, and complex workflows, with many illness episodes unfolding over time and involving encounters with multiple staff members. Clinical and support staff admitted to low confidence in undertaking these encounters and felt they had unmet training needs. New clinical trainees identified several training priorities, including acquiring basic technological skills, becoming proficient in triage, mastering issues such as privacy, consent, and information governance, and developing their communication and clinical skills. Established clinicians’ training priorities included shifting from basic competence to being able to provide an advanced level of communication and support to the patient (for example, building and maintaining rapport, attentiveness, and trust), making complex clinical and operational judgements, ensuring that they understood and worked within the limits of technologies, coordinating multi-professional care in a distributed environment, implementing and embedding new technology supported workflows, and training both patients and fellow staff members. Much current training is didactic and focused on particular digital technologies. Participants valued didactic training to acquire basic competence, but their capability and confidence to make complex judgements were usually acquired through experience, informal discussions, and on-the-job methods such as shadowing. Whole-team training was valued but rarely available. These findings were synthesised into an outline set of competencies and capabilities for clinical staff and those in strategic roles.

### Strengths and limitations

The key strength of this study is that multiple methods and data sources were used reflexively and collaboratively to gain a rich picture of training needs and current provision in UK general practice. By applying theories of adult learning, professional capability, socio-technical learning, and organisational knowledge, we were able to document the significant progress made in developing the digital workforce in primary care and identify areas where further progress is needed. The main limitation is that the empirical work was based only in England, Scotland, and Wales, which limits generalisability of findings (though some training materials were from mainland Europe, the US, and Canada). Another limitation is the very sparse data on electronic consultations, which we suggest should be prioritised for future research. We also highlight that our research on training needs for support staff is ongoing.

### Comparison with existing literature

We are aware of only one published systematic review of training for remote consulting. It identified 14 studies, all of which were evaluations of short courses or single training sessions, classroom-based, and in undergraduate medical or health sciences students (one study also included residents).^41^ The authors of that study found, broadly speaking, that students were more knowledgeable and confident after their training than before it. We identified one article on training needs of allied professionals,^8^ but, to our knowledge, there are no articles in the academic literature on training needs of support staff for remote encounters.

To our knowledge, the present study is the first to have produced outline competencies and capabilities for a multidisciplinary team of primary care staff. Several previous peer-reviewed articles have proposed competencies for remote care but all were secondary care based and uniprofessional, covering internal medicine doctors,^42^ medical students in internal medicine,^43^ paediatricians,^7^ nurses,^44^ and physiotherapists.^45^ All these frameworks focused largely or exclusively on video consultations, which is rarely used in in-hours general practice.^46^

### Implications for policy

The Topol Review^4^ and NHS Digital Academy^3^ have inspired a major effort to develop digital capacity and capability in UK health care. Our findings include some impressive examples of this capacity building in action, with many practices growing in digital maturity and their staff gaining knowledge and confidence through formal and informal training. However, we also identified some issues that policymakers may wish to address.

First, while much current training is geared around new digital technologies, most remote triage and remote consultations occur by telephone, and the only safety-critical incidents we heard about in our interviews were associated with telephone consultations. We suggest that more resources are put into ensuring that both clinical and support staff can — in the words of one interviewee who provides training in telephone encounters — *‘get the basics right’* (Sandra, clinical director of out-of-hours service) for telephone encounters.

Second, while didactic classroom training and self-study e-learning courses have an important place in developing *competencies* for the digital workforce, such acquire-and-transfer methods will take learners only so far. We suggest that existing courses are supplemented with training designed to develop capabilities to make unique, situated, and ethically laden judgements,^28^ and to participate in complex, materially supported, and constrained collaborative routines distributed among organisational staff.^34^ Such socio-material knowledge is best developed through on-the-job training, team training, and opportunities for informal learning through social interaction, case discussions, and storytelling.^28^^–^^30^^,^^47^

Third, and relatedly, our findings suggest that summative assessments such as the RCA and CCT are assessing only a fraction of the competencies and capabilities that a clinician needs to deliver remote and digital services.^29^ Indeed, many safety-critical judgements are made *outside* the clinical consultation since they relate to triage and internal cross-referral, and involve collaboration between (for example) a trainee, a support staff member, and a duty clinician.

Fourth, digital training policies focus disproportionately on individual members of the healthcare workforce.^3^^,^^4^ We suggest they are supplemented with initiatives that reflect the need for a more expansive, team-based element of training.

Finally, we strongly recommend that the learning from existing models of good practice is captured and disseminated. Our empirical findings from out-of-hours primary care (where training for telephone consultations was proactive, systematic, and mainstreamed) were strikingly different from those from in-hours primary care (where clinicians and support staff with varying levels of training were sometimes struggling beyond their competence). Roles such as the ‘clinical coordinator’, a senior clinician who is co-located with staff taking telephone calls and oversees and supports them, should be explored. There is also scope for cross-national learning from examples of good practice that are currently country specific.

In conclusion, this study has found that learning to provide remote general practice services is occurring in a fast-paced and often understaffed context. Where training exists, it is often didactic and technology focused, and, while such formats are appreciated, they do not fully prepare people for the contexts and complexity within which important clinical and clinically related decisions are being taken. The ability to make such decisions appears to be gained primarily through experience, informal discussions, and on-the-job methods of learning. The distributed nature of remote and digital work mean that team training and system learning must be part of the overall training strategy. Training programmes and policies need to reflect these important pedagogical insights.

## Data

The dataset relied on in this article is available from the corresponding author on reasonable request.
